# Combine or Separate Future Pain? The Impact of Current Pain on Decisions about Future Dental Treatments

**DOI:** 10.1371/journal.pone.0064057

**Published:** 2013-05-21

**Authors:** Eduardo B. Andrade, Marco Aurélio Bianchini, Newton Lucchiari

**Affiliations:** 1 FGV, Rio de Janeiro, Brazil; 2 Universidade Federal de Santa Catarina, Rio de Janeiro, Brazil; University of South Australia, Australia

## Abstract

Patients are often given the option of undergoing future painful treatments in one or multiple sessions (e.g., extracting two wisdom teeth on one or two different days). In a randomized controlled field experiment, we investigated the impact of transient pain on patients’ decision to combine or separate future periodontal treatments. The main results show that most patients preferred to have the future treatments take place in one session when they made their choice after a painless examination (i.e., general clinical exam). However, the patients’ preference for combining the future treatments did not differ from chance when the choice was made immediately following a painful examination (i.e., pocketing and bleeding on probing exam). The impact of pain on decision making is observed within and between participants. Current pain seems to lead patients to question their ability to endure future painful treatments in one session.

## Introduction

The literature on emotion and pain has traditionally examined issues related to the measurement of pain [1], the perception and recall of painful experiences [2], the biological properties and alleviating mechanisms of pain [3], the impact of pain on cognitive processes [4], and the impact of patients’ pain on health professionals’ treatment decisions [5]. Interestingly, research from the medical, dental, and decision sciences has been relatively silent on how a patient’s current level of pain influences *his/her own* treatment decisions [6]. This gap in the literature is somewhat surprising given the prevalence of shared decision making in medical contexts [7] and the knowledge that visceral feeling states (e.g., hunger, thirst, and pain) impact judgment and decision making [8]–[9]. This study attempts to fill this gap by investigating the extent to which transient and exogenously induced pain impacts patients’ temporal preferences about future dental treatments.

### Combine or Separate Future Pain?

Patients are often given the option of undergoing future painful treatments either in a single session or in multiple sessions (e.g., a patient who has the option of undergoing multiple cosmetic surgeries in a single versus multiple-visit interventions [10], a dental patient who can choose between a single or multiple-visit root canal treatment [11], or a cancer patient who provides input into his/her radiotherapy schedule [12]). Although a patient’s decision to combine or separate painful experiences has not been investigated, economists and psychologists have examined an individual’s temporal preferences for a sequence of emotionally charged events. Based on prospect theory and the diminishing sensitivity of the loss function [13], it has been proposed that integrating losses reduces the marginal negative impact of the event. As a result, people will tend to combine losses in a single period [14].

The first question that we try to answer is whether the findings obtained in financial and/or hypothetical settings [14], [15] can also be observed in situations where individuals make consequential decisions about future medical treatments. That is, do patients prefer to combine or to separate future painful treatments? The choice to combine may prevail simply because it reduces commuting and scheduling issues, or it may represent the doctor’s preference. In this paper, we test if the preference for the combination of treatments is maintained when these exogenous factors are held relatively constant, either statistically or by experimental design.

### Feeling the Pain While Deciding about Future Pain

The literature has also been silent on whether current feelings can alter the preference for the combination or separation of future negative states. We speculate that an increase in the level of current pain might decrease an individual’s preference for the combination of future pain. Three pieces of evidence support this intuition. First, people tend to exaggerate the extent to which future feeling states and preferences will resemble current ones [16]. Thus, an individual experiencing pain will expect the future unpleasant experience to be more painful relative to an individual who is not experiencing any pain. Second, negative feelings, in general, can lead individuals to form more pessimistic judgments about future events [17]. Third, although preference for the combination of unpleasant experiences is likely, people may spontaneously wonder about their ability to endure a sequence of negative events all at once. When the unpleasantness is perceived as “too large” to be dealt with in a single period, separation is preferred [15]. These pieces of evidence suggest that an individual experiencing pain may be more likely to question his/her ability to cope with the future unpleasant experience all at once, which would in turn reduce his/her preference for combining the future painful treatments.

In the following experiment, we tested (a) if patients display a general preference for the combination of future painful dental treatments, and (b) if higher levels of current pain, exogenously induced by a dental examination, make patients less inclined to undergo the future negative visceral experience in one session.

## Materials and Methods

### Ethics Statement

This study was approved by the review board of the Universidade Federal de Santa Catarina (Brazil). All participants were informed about the general objectives and methods of the study. Written consent was obtained from each participant prior to data collection.

### Participants

The experiment was conducted at a public dental clinic with sixty-two patients pre-diagnosed with periodontal disease in two quadrants of the mouth. The clinic is affiliated with a national university and offers free dental care to the community. Dental patients at this clinic first undergo a pre-examination to identify the type of oral disease, if any, and the recommended treatment. During this screening, the dentist performs a broad clinical exam of the mouth followed by an X-ray. Those pre-diagnosed with periodontal disease (i.e., at least mild inflammation of the gums with some bone loss) are directed to the periodontal disease waitlist. These patients then wait for a second round of more detailed examinations (i.e., level of periodontal disease) and the scheduling of the periodontal treatment. Our study was conducted with pre-diagnosed patients.

### Design and Procedure

The study adopted a 2 [order: painless-painful vs. painful-painless (between subjects)] ×2 [choice: first time vs. second time (within subjects)] mixed design. Each participant indicated his/her preference for future treatment at two points in time: immediately following a painless examination and immediately following a painful examination. The order of the dental examinations was counter-balanced so that participants were randomly exposed to either the painless-painful order or the painful-painless order.

Upon arrival at the clinic, general information about the study was provided, and patients were informed that they were free to decline the invitation or to drop from the study at any point. All patients agreed to participate in the study. They signed an informed consent form, answered a few socio-demographic questions, and underwent two detailed dental examinations meant to assess the level of their periodontal disease. In the *general clinical exam* (i.e., painless condition), the dentist used a mirror as the main instrument to obtain a diagnosis of the patient’s mouth. The dentist assessed the number of teeth, spontaneous bleeding in gingival tissues, presence of intense biofilm and calculus, gingival recession, and halitosis. This exam was expected to produce minimal, if any, pain and discomfort. The other standard examination was the *pocketing and bleeding on probing exam* (i.e., painful condition). During this examination, a periodontal probe (a small dental instrument) was used to measure the sulcus (pocket) between the tooth and the gums as well as to assess any bleeding. In contrast to the general clinical exam, the probe exam was expected to cause some pain and discomfort. Each exam lasted approximately 15 minutes.

Table 1 presents, by treatment (i.e., order of examination), the socio-demographic statistics of the sample, the number of teeth, and the key results of the probing exam (i.e., the probing depth in millimeters and the frequency of bleeding on probing). The statistical analyses show that, as expected, both groups were comparable with respect to their socio-demographic status and the periodontal assessment based on the probing exam. All patients displayed dental elements with probing depth greater than 3 millimeters as well as bleeding on probing–characteristics of periodontal disease.

**Table 1:** Baseline characteristics of the sample.

	Painless-Painful^a^ (n = 31)	Painful-Painless (n = 31)	*P* value
Female, *n* (%)	16 (51.6)	15 (48.4)	>.10^b^
Age, mean (SD)	46.6 (11.1)	47.0 (10.5)	>.10^c^
Monthly income, *n* (%)			
< R$1000	6 (19.4)	10 (32.3)	
R$1001–R$2000	18 (58.0)	15 (48.4)	>.10^b^
> R$2000	7 (22.6)	6 (19.4)	
Education, *n* (%)			
Less than high school	13 (41.9)	14 (45.2)	
High school	10 (32.3)	12 (38.7)	>.10^b^
More than high school	8 (25.8)	5 (16.1)	
Number of teeth, mean (SD)	20.1 (5.8)	21.5 (4.6)	>.10^c^
Periodontal assessment, mean (SD)			
Probing depth (in mm)^d^	2.7 (0.7)	2.6 (0.7)	>.10^c^
Bleeding on probing^d^	1.6 (1.2)	1.4 (1.1)	>.10^c^

a *Painless-Painful* represents patients’ exposure to the painless (clinical/mirror only) examination followed by the painful (pocketing and bleeding on probing) examination. The *Painful-Painless* condition reversed the order of the examinations.

b χ^2^ test.

c *t* test.

d Periodontal assessment was obtained by probing each dental element six times. An average Probing Depth (in mm) per patient was computed by averaging those 6 measurements within and then across available dental elements. An average Bleeding on Probing per patient was obtained by averaging the sum of bleeding on probing of each available dental element.

### Post-Examination Measures

Immediately following *each* dental examination, the patients were asked if they preferred to schedule future periodontal treatments (i.e., scaling and root planing) for a single day or over two days, one week apart. In other words, the patients reported their choice twice and were allowed to change their preference. At the time the patients made their first choice, they were *not* aware that they would have the opportunity to change their mind after undergoing the second dental examination. Because the future treatments would involve both sides of the mouth, they were informed that (a) two identical local anesthetic procedures would be required, and (b) the post-treatment recovery would make it more painful to chew for a few days near the treated regions. They were further told that the actual treatment would take place in a few months.

The dentist explained that the options were identical in terms of clinical outcome, financial costs (i.e., free service), and duration of the surgery. The dentist also stated that the end of the periodontal treatment would be the same independent of the choice of one or two sessions. Therefore, if the participant decided to separate the treatments, the first treatment would occur at Time 1, and the second treatment (or the single treatment option) would occur one week later at Time 2. Additionally, to avoid schedule conflicts, the patients were told that the treatments could be performed after the patients’ working hours.

Following the choice of single or multiple session treatments, the patients reported their current level of pain and discomfort on two numeric rating scales (0 = none; 10 = extreme). After reliability checks, these two items were collapsed to form a pain index–i.e., the manipulation check.

The following is a summary of the experiment protocol: while seated in the dental chair, the patient (a) underwent the first dental examination (painless vs. painful), (b) indicated their preference about the scheduling of the future periodontal treatment and reported their current level of pain and discomfort, (c) underwent the second dental examination (painful vs. painless), and (d) confirmed or revised their preference about the scheduling of the future periodontal treatment and again reported their current level of pain and discomfort. At the completion of all treatments, the patients were asked how much time it took to get to the clinic on average. This variable was used to assess the potential impact of commuting.

Approximately six months later, the patients underwent the periodontal treatment. One day and seven days after the completion of the treatment, a short telephone survey was conducted. The patients were asked to report their current level of pain and discomfort as well as to indicate if they would choose a single or multiple sessions if they had to repeat the treatment (i.e., a measure of regret).

## Results

### Statistical Analyses

Cronbach’s alpha was used to check the reliability of the pain and discomfort items. Analyses of variance and t tests were used to test the impact of the type of exam (painful vs. painless) and the order of examination on the pain index. When the homogeneity of variance assumption was violated, a non-parametric test was used (Mann-Whitney’s U test). Z-tests were used to assess whether the proportion of patients who chose to combine the treatments differed from chance or between conditions. Logistic regressions were used to assess the impact of the pain index and the impact of commuting on the decision to combine or separate future treatments. Within each condition, chi-squared tests were used to compare proportions between subjects and McNemar tests were used to compare proportions within subjects.

### Manipulation Check

The patients’ reported levels of pain and discomfort were strongly correlated [α _after choice 1_ = .90, α _after choice 2_ = .91]. Thus, both items were collapsed to create a single pain index. The pain index showed that the dental examinations produced the expected effects on the patients’ current level of pain. As expected, on the numeric rating scale (10 = extreme), the patients experienced significantly higher levels of pain after the probing exam (*M_painful_* = 4.1, *SD* = 2.5) than after the clinical exam (*M_painless_* = 0.3, *SD* = .82), t(61) = 12.99, *p*<.001). The levels of pain within a given pain condition were the same independent of the order of the dental examinations, *F*<1.

### General Preference

On average, the patients were more likely to combine the future painful treatments (63.7%, *Z* = 3.05, *p*<.005). A logistic regression indicated that the commute time to the clinic did not predict patients’ choice (1 =  combine; 0 =  separate) after the first or after the second dental examination (*χ* ^2^ (1, *n* = 62) = .01, *p*>.10 and *χ* ^2^ (1, *n* = 62) = 1.39, *p*>.10), respectively.

### The Impact of Current Pain

Current pain influenced patients’ preference. After the painless examination, 72.6% of the patients preferred to undergo the future painful treatment in a single session. This proportion was significantly greater than 50%, *Z* = 3.56, *p*<.0005. However, when the decision was made after the painful examination, only 54.8% of the patients chose to combine the future pain in a single session. This percentage was not significantly different from chance, *Z* = .76, *p*>.10. The difference between the two conditions also reached significance, *Z* = 2.05, *p*<.05. Further, a repeated measures logistic regression with the reported pain index as the independent variable and preference as the dependent variable [1 =  combine; 0 =  separate] showed that the patients were significantly less likely to combine the periodontal treatment in a single session as their level of pain increased (*χ* ^2^ (1, *n* = 124) = 6.50, *p* = .01; *β* = −.42, *SE* = .17, *p* = .01). A model with both the commuting time and the pain index as independent variables showed that pain remained a significant predictor (*β* = −.43, *SE* = .17, *p* = .01), whereas commuting time did not (*β* = .02, *SE* = .02, *p*>.10). The omnibus test of the model was significant (*χ* ^2^ (2, *n* = 124) = 6.76, *p*<.05).

An analysis by order of examination (see Table 2) showed that the patients who were exposed to the painless exam first were significantly more likely to choose to undergo the future painful treatment in a single session (80.6%) compared to those patients who experienced the painful exam first (54.8%; (*χ* ^2^ (1, *n* = 62) = 4.72, *p*<.05). The patients who were exposed to the painless exam first were also significantly more likely to choose to undergo the future painful treatment in a single intervention relative to their own preference at time 2 (54.8%; McNemar test, *p* = .02)–i.e., when they then experienced a painful exam. In other words, pain reduced patients’ willingness to undergo the future treatment in one session in both the within subjects comparison and the between subjects comparison. The patients who were exposed to the painful examination first did not change their choice of single or multiple sessions (54.8% vs. 64.5%, McNemar test, *p* = .25).

**Table 2:** Preference for undergoing future painful treatments in a single session.

	Painless-Painful^a^ (n = 31)	Painful-Painless (n = 31)	*P* value^b^
Choice 1, *n*, (%)	25 (80.6)	17 (54.8)	<.05^b^
Choice 2, *n*, (%)	17 (54.8)	20 (64.5)	>.10^b^
*p* value	<.05^c^	>.10^c^	

a *Painless-Painful* indicates that patients made their *first* choice after being exposed to a painless (clinical/mirror only) examination and their *second* choice after being exposed to a painful (pocketing and bleeding on probing) examination. The *Painful-Painless* condition reversed the order of the examinations.

b χ^2^ test.

c McNemar test.

### Post-Treatment Reactions

Only five (out of the 62) patients explicitly asked the dentist to change their choice *during* the actual treatment. Further, in the two *after-treatment* follow-up surveys, the majority of remaining 57 patients reported that they would have made the same choice again. This preference is observed regardless of whether they were asked one day (77%) or seven days (82.5%) after the treatment. Both proportions are significantly greater than chance (*Z* = 4.11, *p*<.0001 and *Z* = 4.91, *p*<.0001, respectively). The high level of consistency with the temporal choice may have been in part due to the relatively low levels of post-treatment pain (*M_day 1_* = 2.2, *SD* = 1.69 vs. *M_day 7_* = .73, *SD* = 1.29; *F*(1, 61) = 62.3, *p*<.0001).

## Discussion

In a randomized controlled field experiment, this experiment demonstrated that people prefer to combine future painful dental treatments. It is reasonable to suggest that the patients’ choice was directly related to their intrinsic desire to endure the future pain in a single session because the financial costs (i.e., free service), duration of the surgery, and date of completion were the same for the one or two session options; the treatments could be performed after the patients’ working hours; and the commute did not influence the patients’ choice.

This preference, however, was moderated by the patients’ current level of pain. As the current level of pain increased due to the dental examination, the preference for the combination of future painful treatments decreased. Our findings are consistent with the idea that pain leads patients to question their ability to endure the future treatment in one session, which increases their willingness to separate the treatments into two sessions. It is worth noting that the patients who were exposed to the painful examination first did not change their minds after the painless examination, which suggests that not only current pain but also *recent* pain may impact patients’ preferences.

Although our research provides direct causal evidence for the impact of current pain on the decision to combine or separate future pain, it does not provide direct evidence for the underlying psychological mechanism(s). For example, the dentist stated that the date of completion of the periodontal treatment would be the same independent of choice. Therefore, if the participant decided to separate the treatments, the first treatment would happen one week in advance of the second treatment. It is possible that patients in the painful (vs. painless) treatment chose to separate the treatments to reduce the dread associated with the waiting [18]. Although this suggestion is possible, there are at least two reasons that make this explanation less likely. First, feelings of dread are often associated with negative events that will occur in the near future, for example, an upcoming electrical shock [19]. The knowledge of negative events occurring in the distant future, which was the situation for our patients, should be less likely to cause feelings of dread. Furthermore, even if feelings of dread were present, separating the periodontal treatment sessions confirms an earlier *beginning* of the negative experience but not an earlier *end* of the experience. In other words, any feelings of dread would still be present in the interval between the first and the second treatment. However, the impact of pain on feelings of dread and subsequent choices represents an interesting research avenue, which, to the best of our knowledge, is yet to be investigated.

In addition, we suggest three other avenues for future research. First, it was not clear from this study whether higher levels of pain at the time of the decision led people to overestimate the future pain, to question their coping skills, or a combination of both. Second, research on the relationship between pain and treatment schedules in other medical contexts (e.g., cosmetic surgeries) is necessary, as is an exploration of the theoretical ramifications of similarities or differences across contexts. Finally, our research was limited to the impact of transient and exogenously generated pain. In the painless condition (i.e., after the general clinical examination), the patients reported no pain at all, which indicates that the patients were not experiencing chronic pain. Because chronic pain may add a new motivational component (i.e., a desire to find relief), it is not clear how this might interact with patients’ tendency to separate future treatment sessions.

In conclusion, this paper focused on a class of visceral experiences (transient and exogenously generated pain) and a particular medical context (dental pain and treatments) in order to provide insight on how visceral experiences influence a patient’s temporal decision making. Due to the amount of shared decision making in medical settings and the fact that patients may be experiencing different emotional and visceral states at the time of such decisions, there is a wide array of opportunities for further research.
